# Experience with bruxism in the everyday oral implantology practice in the Netherlands: a qualitative study

**DOI:** 10.1038/s41405-018-0006-4

**Published:** 2018-11-09

**Authors:** Magdalini Thymi, Annemiek Rollman, Corine M. Visscher, Daniel Wismeijer, Frank Lobbezoo

**Affiliations:** 1 Section of Oral Kinesiology, Department of Oral Health Sciences, Academic Centre for Dentistry Amsterdam (ACTA), Amsterdam, The Netherlands; 2 Section of Oral Implantology and Prosthetic Dentistry, Department of Oral Health Sciences, Academic Centre for Dentistry Amsterdam, Amsterdam, The Netherlands

## Abstract

**Objective:**

To explore how bruxism is dealt with by accredited oral implantologists within daily clinical practice.

**Materials and methods:**

Nine semi-structured interviews of oral implantologists practicing in non-academic clinical practices in the Netherlands were performed, and thematic analysis was conducted using a framework-based approach.

**Results:**

Oral implant treatments in bruxing patients were a generally well-accepted practice. Complications were often expected, with most being of minor impact. Contradictive attitudes emerged on the topic of bruxism being an etiologic factor for peri-implant bone loss and loss of osseointegration. Views on the ideal treatment plan varied, though the importance of the superstructure’s occlusion and articulation features was repeatedly pointed at. Similarly, views on protective splints varied, regarding their necessity and material choice. Bruxism was diagnosed mainly by clinical examination, alongside with patient anamnesis and clinician’s intuition. There was little attention for awake bruxism.

**Discussion:**

Bruxism was generally not considered a contraindication for implantological treatments by accredited oral implantologists. Views on the interaction between bruxism and bone loss/loss of osseointegration varied, as did views on the ideal treatment plan.

**Conclusions:**

There is a need for better understanding of the extent to which, and under which circumstances, sleep and/or awake bruxism can be seen as causal factors for the occurrence of oral implant complications.

## Introduction

Clenching and/or grinding of the teeth is a characteristic expression of bruxism and can occur while sleeping and/or while being awake.^1^ In the field of restorative dentistry, bruxism is traditionally dealt with as “the bad guy,” to be associated with various types of failures of dental restorations.^2^ Regarding dental implants, it has been suggested that bruxism can lead to technical, and to a lesser extent to biological, complications even if, to date, no prospective evidence exists to prove this.^3^ Alongside, but also as a consequence of this research paucity, no evidence-based gold standard exists about the optimal way to treat bruxers with dental implants. So far, clinical recommendations concerning occlusion and articulation as well as material choices are given in the form of expert opinions.^4,5^ Consequently, a variability in treatment approaches of bruxing patients can be expected amongst dentists placing and/or restoring dental implants.

As for detecting the presence of sleep bruxism (SB), a polysomnographic study of sleep with audio-visual recordings (PSG-AV) is recommended to set a definite diagnosis.^2^ This method is not suitable for the daily dental practice.^2^ On the other hand, the much more feasible methods of self-report and clinical examination lack the validity of a PSG-AV, and can only indicate the presence of possible and probable SB.^1^ Portable electromyographic (EMG) devices seem promising alternatives of PSG methods, although their widespread implementation in the dental practice is still in an initial phase.^2^ Likewise, self-report and clinical examination can at best only lead to the diagnosis of probable awake bruxism (AB).^1^ Therefore, accurately diagnosing both circadian manifestations of bruxism (i.e., SB and AB) is still a significant challenge faced in everyday clinical practice.

As a result of the above, the question arose of how bruxism is actually dealt with in the context of the clinical, non-academic reality of the oral implantology practice. It was hypothesized that experienced practitioners will have had to deal with all kinds of aspects of implant treatments in bruxing patients: from identifying the condition of bruxism to the planning and outcome of their treatments, including possible complications related to the condition.

Furthermore, it was hypothesized that experiences with bruxism derived from daily practice could be a rich source of information for academia, meaning, investigating what works in real life, and what does not, can be a guide to defining future research questions and study protocols driven by clinical pragmatism.^6^ In turn, as high-quality research in the field of bruxism and implant complications will undoubtedly emerge in the future, it is important to gain insight into the present status of clinical practice, as an aid to the design of reality-motivated implementation policies of research results.^6^ Therefore, the aim of this study was to explore and critically analyze the attitudes and experiences acquired by experienced oral implantologists when dealing with bruxing patients in a non-academic setting.

## Methods

### Study design

An effective way to gather a broad spectrum of information related to personal attitudes and experiences is the use of semi-structured interviews, which are a form of qualitative research.^7^ Thereby the researcher/interviewer can acquire in-depth information from the interviewee on pre-conceived topics, while new, unthought of ideas are allowed to emerge and be explored.^7^

### Interviewee sampling

Purposive sampling, that is, sampling based on a specific criterion, was used to select interviewees.^8,9^ The criterion was that implantologists should have considerable experience in performing oral implant treatments. Therefore, only dentists accredited to perform such treatments by the Dutch Association of Oral Implantology (Nederlandse Vereniging voor Orale Implantologie, NVOI) were selected for inclusion, since this group comprises of profoundly experienced professionals in the field of oral implantology in the Netherlands. Alongside, it was aimed to recruit implantologists from geographically spread areas of the Netherlands in order to account for possible socio-geographical influences.^10^ After permission of the NVOI, the accredited implantologists were alphabetically invited to participate by an e-mail with a short, standardized text, accompanied by a brochure describing the purpose and methods of the study. Implantologists were informed about the interviewer’s professional background and the motives to perform this study. When an implantologist agreed to participate, no other implantologists practicing in the same area were contacted.

### Interview conduct and data analysis

Semi-structured interviews of approximately 30 min with dentists-implantologists who run a practice focused on the placement and restoration of dental implants in the Netherlands were conducted by M.T. M.T. is a dentist, trained and clinically active in the field of orofacial pain, oral movement disorders, tooth wear, and dental sleep medicine, and a PhD student at the Academic Center for Dentistry Amsterdam (ACTA). M.T. does not perform oral implant treatments. Training for the conduct of the study included self-study and the performance of three pilot interviews, under the supervision of a researcher (A.R.) with training and experience in the field of qualitative research. A.R. furthermore is a physical therapist, active as a clinician and academic in the field of orofacial pain and oral movement disorders. The interviewer had no personal or professional affiliation with the participants. All interviewed implantologists were informed about the purpose and methods of the study and the professional background of the interviewer, and gave a written informed consent. The study was reviewed and approved by the Ethics Committee of ACTA (reference number 2016016).

Prior to the first interview a number of relevant domains were defined, and subsequently, an interview topic guide was designed.^9^ The goal of the topic guide was to function as an agenda and memory aid for the interviewer, in order to ensure systematic collection of information on the predefined domains.^9^ These domains were defined based on the available literature, the feedback from the three pilot interviewees, and the professional experience of an expert panel consisting of the co-authors of this paper (M.T., A.R., C.M.V., D.W., F.L.). C.M.V. is a physical therapist, active as a clinician and academic in the field of orofacial pain and oral movement disorders. F.L. is a dentist, active as a clinician and academic in the field of orofacial pain, oral movement disorders, tooth wear, and dental sleep medicine. D.W. is a dentist, active as a clinician and academic in the field of oral implantology. A.R., C.M.V., F.L., and D.W. are also actively involved in undergraduate and postgraduate education of dentists in the Netherlands. The domains covered were: feasibility of, and experiences with implant dentistry in bruxing patients; attitudes regarding the features of an implant treatment plan in bruxing patients; attitudes regarding the diagnosis of bruxism in the clinic, and attitudes related to scientific research in the field of implant dentistry and bruxism (Table 1).

**Table 1:** Main domains included in topic guide

Main domain	
1.	Feasibility of, and experiences with implant dentistry in bruxing patients
2.	Attitudes regarding the features of an implant treatment plan in bruxing patients
3.	Attitudes regarding the diagnosis of bruxism in the clinic
4.	Attitudes related to scientific research in the field of implant dentistry and bruxism

These four domains were explored during the interviews using open-ended questions, and interviewees were encouraged to bring up relevant items during the conversation, even if they were not included in the topic guide.^7,9^ Data collection and analysis occurred concurrently, allowing for new themes to be fused into the topic guide as the study progressed.^9^ The interviews took place at a location of the interviewee’s choice, which in all cases was their dental practice. Only the interviewer and interviewee were present during the interview. At the start of each interview, interviewees completed a questionnaire on demographic data, and data related to their education (year of birth, year of graduation, place of studies, year of registration as an oral implantologist in the Netherlands, postgraduate education in oral implantology and/or bruxism, and the approximate number of implant-borne superstructures placed per year, that is, 10–50, or ≥50).

Each interview was audio-recorded, and thereafter, a verbatim transcription was made, with any information revealing the identity of the interviewee removed. The transcriptions were not returned to the interviewees for comments or corrections, and no interviews were repeated. Thematic analysis of each transcript was performed by M.T. shortly after its acquisition, using a framework-based approach.^9,11^ This analytic method was carried out in successive steps.^9^ First, each transcription was investigated line-by-line for the identification of initial themes. Initial themes were given short, descriptive titles. Their identification was based on published literature, the data acquired from the pilot interviews, and the professional judgment of the investigator (M.T.). Next, conceptually related initial themes from the available interviews were grouped into main themes, each of which consisted of sub-themes. This process was done by hand, without the aid of data-analysis software. The analysis was reviewed by A.R., and any disagreements in the interpretation of the data were resolved by discussion. Stepping backwards in the analytic process was allowed, when newly occurring initial themes from subsequent interviews required separate main or sub-themes, or when deeper familiarization of the researchers with the data led to new insights for their grouping. Interviews continued to be performed until saturation, that is, until no new main or sub-theme emerged out of the data. After this, two more interviews were performed, in order to confirm saturation. Once saturation was achieved, the main and sub-themes were given numerical codes. These codes were used to label the transcribed data, that is, to assign the interview texts to the proper main and sub-theme. Next, a thematic chart was created in Microsoft Excel 2010 software, in which the top row represented the main themes, under which each sub-theme was given a column. These columns served for clustering of all textual data that were related to the respective sub-theme. The textual data in this step were summarized, that is, their essence was extracted with care not to lose the context or language in which they were expressed. This allowed for the synthesis of data and formulation of conclusions per sub-theme, and subsequently per main theme.^9^

## Results

### Participants

**Fig. 1:**
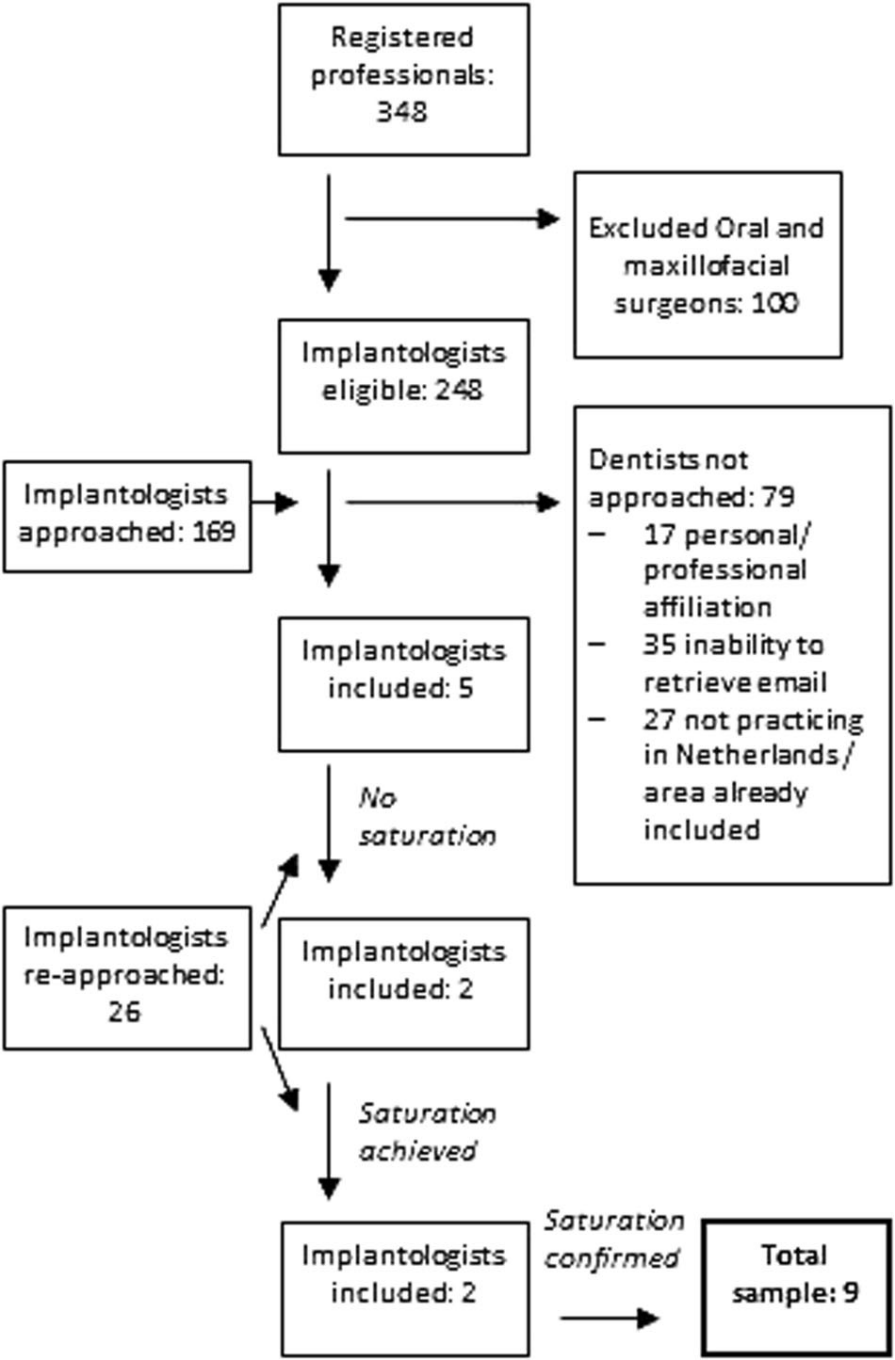
Inclusion flowchart

In August 2016, 348 professionals were registered as NVOI-accredited oral implantologists. This number includes 100 oral and maxillofacial surgeons, who were excluded from our selection procedure, since they are mainly involved with the surgical, but not the prosthetic part of implant treatments (Fig. 1). Between the 26th of August and the 22nd of September 2016, 169 dentists (19 females, 150 males) were approached for participation. The other 79 dentists were not approached for the following reasons: personal or professional affiliations with the interviewer,^12^ inability to retrieve the e-mail address,^13^ not practicing in the Netherlands, or inclusion of a dentist practicing in the nearby area.^14^ After this recruitment round, five dentists agreed to participate. Three implantologists reported not being able to participate in the study due to lack of time, while the remaining 161 did not provide a reason for non-participation. Since saturation was not achieved after these five interviews, a second round of recruitment was performed between the 29th of September and 11th of November 2016. During this period, an e-mail reminder was sent to a selection of 26 implantologists practicing in areas of the country from where no one had been interviewed before. As a result of this recruitment round, two more interviews were performed, after which saturation of data was achieved. From the same recruitment round, two more interviews were performed, confirming the saturation of data. Thus, in total, nine implantologists were interviewed in this study, between August and December 2016.

All interviewees were male, with a mean (range) age of 49 (33–59) years (Table 2). The nine interviewees were established in 5 out of the 11 provinces of the Netherlands, in various distances from academic dental institutions. The mean (range) number of years of practicing dentistry was 23 (10–31). Interviewees had acquired their dental degree in the Netherlands. All interviewees reported having followed postgraduate education in the field of oral implantology in the past 5 years, in the form of courses, lectures, congresses, and/or reading professional literature. Six interviewees reported having followed postgraduate education related to bruxism.

**Table 2:** Sample characteristics

Total sample size	9
Male/female	9/0
Mean (range) years of practicing dentistry	23 (10–31)
Mean (range) years of being an accredited oral implantologist	10 (2–18)
Approximate number of implant-borne superstructures placed per year	10–50: 1 participant, ≥50: 8 participants
Number of participants having followed postgraduate education in the field of oral implantology in the past 5 years	9
Number of participants having followed postgraduate education in the field of bruxism in the past 5 years	6

### Thematic analysis

Out of the thematic analysis, four main themes emerged, which coincided with the four domains that were described by the expert panel prior to the interviews: (1) bruxism and implant treatment outcomes; (2) treatment aspects of implantological interventions; (3) diagnosis of bruxism; and (4) improvement of care in the future. Each theme consists of several sub-themes. The themes and sub-themes are described below and translated quotes from the original transcripts in *Italics [interview number]* are provided when useful. Within the quotes, text within parentheses (…) is inserted when needed to provide the reader with contextual information. A comprehensive overview of the results is provided in Table 3, with the most important findings described in the text below.

**Table 3:** Main themes, sub-themes, and summary of experiences and attitudes

***1. Bruxism and implant treatment outcomes***
1. General attitude about impact of bruxism on oral health
•Bruxism is damaging (wear, endodontic treatments, tooth loss, fractures, pain, or limitation of movement) •Without pain function is not impaired •Occlusion/articulation are important mediators for damaging effects
2. Feasibility of implant dentistry in bruxers
•Positive attitude: implants are possible, bruxism is not a contraindication (unless there is pain, some precautions needed, it can even help distribute forces better, better than conventional porcelain) •Negative attitude: clenching can be dangerous, possible, but with uncomfortable feeling for dentist
3. Encounters with complications
Variation in attitudes: •Occurrence of complications: never, there is always something, real bruxers will break everything, no control over when it goes well/sometimes miraculously well, no complications until occlusion changes over time due to wear of all teeth except the implant-borne restoration •Type of complications: usually chipping of porcelain, wear or fracture of FP, wear or fracture of mesostructure, wear of antagonists or problems with antagonistic porcelain, fractures of screws, fractures of implants, bone loss •Bone loss: not possible, only after infection, independent of infection •Loss of osseointegration: possible, impossible
4. Mechanism of complications
Bone loss/loss of osseointegration: •Excessive loading can lead to bone loss, which can be followed by bacterial invasion, ultimately leading to peri-implantitis •Inflammation pre-exists and subsequent overload can lead to more profound bone loss •Load can cause micro-movements of the implant in the bone, which can lead to loss of osseointegration •Load can cause loss of osseointegration, only if this was poor already •Peri-implantitis occurs mainly due to other reasons (e.g., wrong placement of implant, cement remnants) •Uncertainty about form of relation Other complications: inattentiveness of dentist (tightening of screws, occlusion, etc.), materials, wear and subsequent change of occlusion over time, bad starting point (e.g., after peri-implantitis treatment), technician
5. Consequences and treatment of complications
•Chippings: usually not very troublesome, investigate cause •Finances: reparation under warranty, pain less with cheaper materials, burden for practice is low •Emotional: irritation for patients, blame on dentist, burden not high for dentist •Practical issues: immediately new implant after removal of fractured one, harder suprastructure materials may lead to other, deeper problems, time investment
***2. Treatment aspects of implantological interventions***
1. Assessment of patients
•Thorough investigation of signs of function in every patient from the start of therapeutic trajectory •Understand why fractures occurred in the past •History/knowing the patient/intuition helps •Make intraoral pictures and discuss them with patient
2. Treatment features
•Occlusion, various views: only when biting hard in maximal occlusion, out of occlusion, can make contact, may be out of contact, check at preventive check-ups •Articulation: no contact if lateral forces are anticipated, strive for front and canine guidance, may be out of articulation, check at preventive check-ups •Protection: (a) Splint: is important, not so much (b) Splint material: soft splint gives more compliance, hard splint is less comfortable, hard splint is comfortable, (c) Splint design: should allow freedom of movement, thin (d) Advices and awareness regarding bruxism during the day •Materials/technical issues, variety of views: diameter, strength, number of implants, implants blocked, bone augmentation, occlusal pattern, material of crowns, technician skills, informed consent, advices to referring dentist •Removable prosthesis: (a) Concept A: as much as possible mucosally supported so that pain is felt when bruxing, bite not too high, lingualized bilaterally balanced occlusion (b) Concept B: strong basis with soft teeth, teeth wear, and are replaced, basis does not break (c) Taken out during sleep
3. Communication with patients
•Discuss beforehand: risks/expectations, protection, FP out during sleep, written informed consent •Awareness of problem: pictures, feel the fremitus, discussion in order to increase acceptance, so that blame will not be put on dentist, increase compliance with advices, some are already aware •Discussion may come across with denial or intervention with private issues
4. Role of general practitioners
•Important for longevity of implant-supported restorations •Should pay more attention to occlusion and articulation when placing suprastructure •Role of preventive check-ups for early detection in occlusion and articulation changes •Communication about materials/protection/advices for canine guidance •Complications due to improper implant component handling
5. Sources of information
•Literature, courses, undergraduate education •Experience, intuition
***3. Diagnosis of bruxism***
1. Importance of diagnosing bruxism
•Very important, should be part of routine •It would be nice to know •Not per se recognizing bruxism, but in general being able to discover the cause of failures is important
2. Diagnostic approaches
•Extraoral examination: shape of face/muscles, activity of jaw, general appearance/temper •Intraoral examination: tooth wear, presence or history of fractures, endodontic treatments, mobility, furcation problems, cheek lines, lost teeth, type of bite (deep/open) •Anamnesis: self-report, partner report, temporomandibular joint complaints •Other: “a feeling,” knowing the patient, experience
3. Challenges
•Uncertainty about diagnosis: •Importance of intraoral and extraoral signs? (validity) •Importance of self-report? (patients not aware, denial for the sake of not taking responsibility/financial aspects, privacy issues) •What is the definition of a bruxer, how do you know if someone is currently active
***4. Improvement of care in the future***
1. Role of education
•Attention of general practitioners for occlusion and articulation, learn how to see signs of bruxism and take it into account during treatment planning
2. Role of diagnostic approaches of bruxism
•It is important: treatment should be based on good diagnosis, improve compliance of wearing protective splint, difficult since bruxism can fluctuate, simple chair-side tool, device for home, referral clinic for extreme cases •Not important: complications mainly due to infection, constructions already strong enough for everyone (bruxers and non-bruxers)
3. Role of treatment approaches of bruxism
•Does not seem to be an important issue for implantologists, but may be for dentistry in general •Use of botulinum toxin
4. Other issues
•Define who is a bruxer •Information brochures regarding bruxism/more understanding from patients •Other/improved materials •No reason for further research •Splint features

#### Theme 1: Bruxism and implant treatment outcomes

(1) *General attitude about impact of bruxism on oral health*: Interviewees considered bruxism damaging for the dentition, as it can cause tooth wear, fractures of teeth and restorations, endodontic pathology, loss of teeth, pain, and limitation of mandibular movement. Occlusion and articulation were viewed as important mediators of the damaging effects of bruxism. *“I think that bruxism, that it is important that people do not do this, it just damages your dentition” [3]*. On the other hand, it was also mentioned that in the absence of pain, adequate oral function is not limited by bruxism.

(2) *Feasibility of implant dentistry in bruxers*: There were two opposite attitudes regarding the feasibility of implant dentistry in bruxing patients, a stronger, positive attitude as opposed to a less prominent, negative one. The first advocates that bruxism is generally not a contraindication for implant dentistry. Some precautions need to be taken though, for example, interviewees argued that the patient should be without orofacial pain prior to the start of implant treatment, and the condition should be taken into account during treatment planning. From the interviews, the image arose that implant-related interventions could even be helpful for the bruxer with multiple tooth or teeth loss, since the exerted forces would be better distributed over the dentition. *“With implants you can of course help distribute the forces better, when you have many lost teeth and you have an occlusion on 4 or 5 teeth… this is also not helpful for the (intraoral) situation” [4]*. On the other hand, some expressed negativity and an uncomfortable feeling about implant treatments in bruxers, especially when it came to clenching activity.

(3) *Encounters with complications*: There was a wide variation in the experiences and attitudes regarding the occurrence and type of implant complications related to bruxism. One interviewee had never experienced any complication directly related to bruxism. In general, though, complications were expected by the interviewees, either sometimes or, for the “real bruxers,” always. *“Sometimes you will experience some things, yes, and I have to say that, in implantology, it is not that bad, but if it goes wrong then it is often very annoying” [6]*. Chipping of porcelain was mentioned as the most common complication. Other types of complications were: wear or fracture of a full removable prosthesis (RP), wear or fracture of a mesostructure, wear of antagonists or problems with antagonistic porcelain, fractures or loosening of screws, and fractures of implants. Divergent experiences and visions emerged regarding bone loss and loss of osseointegration as a consequence of bruxism. A similar lack of consensus was found for loss of osseointegration due to bruxism, with some having experienced it, while others arguing it is not possible (see paragraph below). *“An implant that loosens due to overload I do not believe in, that is an implant that was never well integrated” [9].*

(4) *Mechanism of complications*: Factors mentioned as being related to complications of implant treatments are the inattentiveness of the restoring dentist (e.g., insufficient tightening of screws or control of occlusion), material/implant properties, tooth wear and subsequent change of occlusion over time (leading to more contact of the superstructure with the antagonists), having a bad starting point (e.g., bruxing on an implant construction with a high crown-to-root ratio), and skills of the technician. Various views on how bruxism could be related to bone loss, peri-implantitis, and loss of osseointegration were expressed:

- Excessive loading can lead to bone loss, which can be followed by bacterial invasion, ultimately leading to peri-implantitis. *“More and more voices are raised to support that peri-implantitis has at its origin a mechanical component, tension, then you get bone loss, and after that you get the bacterial invasion and everybody starts calling it peri-implantitis, because it is inflamed” [2]*.
- Inflammation pre-exists and subsequent overload can lead to more profound bone loss. *“I do not believe that overloading can cause bone loss, but overloading can cause bone loss when there is an underlying infection” [3]*.
- Load can cause micro-movements of the implant in the bone, which can lead to loss of osseointegration.
- Load can cause loss of osseointegration, only if this was poor already.
- Peri-implantitis is mainly caused by other factors, for example, cements remnants.
- Uncertainty about possible relationship between bruxism and peri-implant bone loss.

(5) *Consequences and treatment of complications*: Porcelain fractures were generally not considered as troublesome complications. Smoothing out the edges was usually sufficient, though in more severe cases the superstructure may need to be replaced. When such fractures occur, it is a good moment to pause and investigate the cause of it, and think of preventive measures. *“Is it (chipping of porcelain) a disaster? Oh well, you fix it, but then it is a moment to check with attention, may be should I, for example, build up the canines?” [4]*. Material-wise, it was argued that a cheaper material, such as composite, may cause less financial pain when needing to be replaced, than a more costly ceramic material. Also, it was mentioned that when using harder materials for the superstructure, such as zirconia for fixed dental prostheses (FDPs) or metal parts in RPs, the occlusal forces may be led to more distant components of the implant, and other, “deeper” problems may occur. *“The solution to use full zirconia is nice, I always say, but you should realize that when you make the crown constantly harder, in the end either way something will break, and then it will break deeper” [5].* Complications in general could have a negative emotional impact on patients, who could become frustrated for having to visit the implantologist too often, and put blames on the implantologist. Some implantologists were not fond of the issue of complications in bruxers, but in general this did not appear to be a significant problem, neither emotionally, nor financially.

#### Theme 2: Treatment aspects of implantological interventions

(1) *Assessment of patients*: In the context of treatment planning, implantologists found it generally important to pay close attention to intraoral signs of heavy mandibular function from the start of the therapeutic trajectory. Taking time to observe signs of function, such as wear facets, was important, and implantologists should similarly try to understand why certain teeth may have fractured in the past. Making intraoral photographs and discussing them with the patients was considered as extremely helpful. Knowing the patient for a long time, but also sensing that something is going wrong in the mouth was also mentioned as a source of information. *“There is no strict protocol, that one is (a bruxer), that one not…you sense things, you think something here is not going well” [7].*

(2) *Treatment features*: Implantologists gave ample attention to the topic of the features that their implantological treatments should have in (presumable) bruxers. Their views were concentrated around aspects of occlusion, articulation, protection, materials, and other technical issues.

Paying attention to occlusion on FDPs was considered as very important, and the matter was approached in several ways: (a) superstructure occluding with the antagonists only when biting hard in maximal occlusion, (b) superstructure completely out of contact, and (c) superstructure allowed to stay in regular occlusion. Opinions regarding articulation were pointing more towards one direction: interviewees generally agreed that superstructures should be free from contact with antagonists during lateral movements of the mandible. If the superstructure does participate in a lateral movement, then support from neighboring teeth in the form of group guidance is preferred. Besides adjusting the superstructure itself, a common way to achieve these goals is by building in canine guidance on non-implant-borne superstructures with, for example, direct composite restorations. Occlusion and articulation should be controlled during the preventive check-up visits at the general dentist, because, interviewees agreed, the dentition not only wears, but is also in slight movement during the course of years. Consequently, over time a, superstructure may acquire undesirable contacts with its antagonists.

Various views were expressed about the need for protection of the final restoration(s). Making an occlusal splint to wear during sleep was always advised by some interviewees, while others argued that a protective splint in necessary in some, but not all cases of suspected bruxism. No specific criteria were given as guides for deciding in which cases a splint should be made. As for the material, some preferred a hard and others a soft one, for reasons of patient’s comfort. *“I don’t really feel it makes such a difference, to be honest (in protective effectiveness of a soft or hard splint), I find it important that they wear it, that is why I choose a soft one” [4].* Less attention was paid to AB, with only one brief mentioning of giving advices and making the patient more aware of it.

There were plenty of views on the characteristics of materials that can or cannot be used when bruxism was expected. Interviewees preferred wide implants, and if necessary, a bone augmentation should be performed for creating space for a wider implant. A longer waiting period should be kept before loading an implant in an augmented site. As many as possible implants should be placed and neighboring implants should be blocked. The skills of the dental technician were important for the longevity of restorations. Interviewees had different preferences for FDP materials, such as metal occlusal surfaces, monolithic zirconia, lithiumdisilicate, or composite. On the other hand, some argued that in terms of material choice, treatment is the same, regardless of whether or not someone bruxes.

When an RP is made, this should be taken out during sleep. Two interviewees mentioned following a specific pattern when making full RPs in bruxing patients. One strives for a prosthesis design that as much as possible is mucosally supported, so that people will feel pain when bruxing. *“We go as resilient and mucosal supported as possible, so that people will really have pain at the moment they start to grind” [2].* Another interviewee choose for a prosthesis supported by rigid metal parts and provided with soft artificial teeth. Due to its metal support the prosthesis will not break, but the teeth are allowed to wear. When worn, the teeth are to be replaced.

(3) *Communication with patients*: Bruxism and its consequences for implant treatments were discussed with patients. Prior to the start of the treatment, patients were informed about the risks for the course of treatment (e.g., superstructures may wear fast), and expectations regarding future needs (e.g., worn denture teeth that may need to be replaced periodically). Patients were also informed that their future dentures need to be taken out during sleep, or on the need to wear a protective splint. By some, all this information was put in a written informed consent.

In order to increase the awareness of patients on their bruxing behavior, interviewees took time for discussion, used intraoral photographs, and had the patients feel the fremitus of their teeth (i.e., the movement of teeth when subjected to functional occlusal forces^15^). By raising awareness, patients might be more inclined to accept possible future complications, not put the blame of their occurrence on the implantologist, and be more compliant with preventive advice given. Some patients are already aware of their bruxism, others are not, but do recognize it after discussion, while some remain reluctant to accept they might be bruxing. *“The art is to make the problem very clear to the patient…there are people that (say) ‘I do not grind’, but when you show them the (dental wear) facets, then of course they start to see it themselves” [4]*.

(4) *Role of general practitioners*: General dentists were the main professionals that refer patients to the interviewed implantologists. Often, they place the superstructure after the implant has been placed by the implantologist. Some interviewees reported that general dentists should pay more attention to proper occlusion and articulation of the superstructure they place, a matter which is often overlooked. Interviewees communicated with the referring dentists, and advised them on matters such as the need for creating canine guidance, the need for protection in the form of a splint, or on material choices. These advices are not always followed, either due to reluctance of the general dentist or of the patients themselves. *“Then I put in the letter to the dentist to consider placing a splint for the night, which dentists never make, because they think this is nonsense, they say that the patients will not wear it anyhow” [3]*. Furthermore, the general practitioners could play an important role after the implant treatment is complete, by signaling changes in occlusion and articulation during the regular check-ups. What also emerged from the interviews is that general dentists’ improper handling of implant components might, in some cases, be an important source of complications, irrespective of bruxism (e.g., when abutment screws are not tightened properly).

(5) *Sources of information*: Information on which decision-making is based in practice was collected from various sources. Interviewees mentioned postgraduate courses, reading literature, but also their own experience and intuition. Undergraduate education was not referred to as an important source of knowledge. *“Everything that has to do with cantilevers (I do not make)…that is based on my feeling, on nothing else” [2]*.

#### Theme 3: Diagnosis of bruxism

(1) *Importance of diagnosing bruxism*: Opinions about the importance of diagnosing bruxism in the implantologist’s practice diverged. At one end, it was mentioned that knowing whether or not the patient bruxes is very important and should be investigated routinely. *“One (dentist) pays more attention to it (bruxism) than the other, and the other pays more attention to other things…I think that it is a part of… you look at several issues: the condition of the dentition, how is it restoratively, caries sensitivity, periodontally, and this is also how you should look functionally, what is someone doing with their dentition” [7]*. At the other end, it was mentioned that bruxism occurs in virtually everyone, and it is not important per se to know if someone is active, but instead to make an effort to understand the multiple reasons of why things (restorations, teeth, etc.) fracture or otherwise fail in the mouth.

(2) *Diagnostic approaches*: It was not a topic of controversy that interviewees used intraoral and extraoral examination, and to a lesser extent patient anamnesis, in order to collect signs and symptoms that indicate the presence of bruxism. There was, however, variability in the signs and symptoms examined. Extraoral examination involved observing the shape of the face and size of visible masticatory muscles, the activity of muscles while talking, and the overall impression that the patient gives, in terms of their temperament. *“The character of people, how they come across, a couple…how they dress, how they present themselves, you can see if they are controlling biters or relaxed people…so that gives me a suspicion” [2]*. Intraoral examination commonly involved looking for tooth wear. Current, or history of, fractured teeth or restorations is important, as well as the presence of many, otherwise unexplained, endodontic treatments and lost teeth. *“Then you look at wear facets and also the teeth that were lost, because the history often tells a whole story, if at one side I have many endodontic treatments and the other side not…possibly because the forces on that side were higher” [4]*. Teeth may show an increased mobility or fremitus, molars could have furcation problems that are not explained by an overall periodontal disease, and hyperkeratotic cheek lines may be present. A deep anterior bite mentioned being associated with clenching, and a more open bite with grinding. The anamnestic part, where patients are asked whether they recognize bruxism, was not considered trustful, as it was mentioned that many are unaware of their activity, though their bed partners may sometimes be. Temporomandibular joint complaints were sometimes also used as indicators of bruxism. Additionally, some also used their experience and an intuition as aids to detect bruxers.

(3) *Challenges*: Interviewees struggled with some issues when attempting to diagnose bruxism. Intraoral signs and self-report were not considered watertight diagnostic methods. Acquiring a partner-reported diagnosis brought some dentists in an uncomfortable position, since it involved asking patients questions that might intervene into their private life. Another issue that emerged is related to the mere definition of a bruxer, that is, when is one defined a bruxer and how is the time-variant nature of bruxism dealt with? *“Then the first question is, of course, what is a bruxer, where is the limit, it is a very big grey area, this is the difficult thing about it” [5].*

#### Theme 4: Improvement of care in the future

(1) *Role of education*: As for which component in the education of dentists could help improve the care for bruxing patients, there was a focus on the importance of proper occlusion and articulation, since it may prevent future complications. Furthermore, general dentists should learn to at least see the signs of bruxism, and to take this into account when planning their treatments. *“I don’t know if you can stop bruxing, but I think there should be more attention to in the education, to learn how to see it…if you think that someone is bruxing that you build in a situation in the mouth, that you protect many things a bit more, there has to be more attention for this, it does need some time, but it is the neglected child in the undergraduate dental training” [4].*

(2) *Role of diagnostic approaches of bruxism*: For some, improvement of bruxism diagnostic methods was important, since now some treatment plans are based on a suspicion, rather than a solid diagnosis of bruxism. Having, however, a solid diagnosis should be the basis on which a subsequent treatment plan is built. Also, if bruxism could be objectified, patient compliance with wearing a splint during sleep could increase. On the other hand, it was also argued that improving the diagnostic methods is not necessary. This was either because the complication rates in bruxers are already very low or because implant constructions are made as strong as possible, regardless of whether or not someone might be bruxing.

If diagnostic methods were to be improved, their main feature should be simplicity in use. The example of a chair-side screening tool was given, allowing the dentist to track changes of the oral situation indicative of bruxism during the periodic preventive check-up, and guiding the decision to use a more thorough diagnostic method. Also, a device that patients could easily use at home, while sleeping, and which could objectify bruxism activity was suggested. *“There should be an objective test indeed (to know) if someone is a bruxer or not, but then you have the very severe ones and the lesser, and the others that do it once per month, and that one every night, it is, I do find it difficult” [3].*

(3) *Role of treatment approaches of bruxism*: There was little focus of implantologists on the topic of actually treating bruxism. Using botulinum toxin for this purpose was considered.

(4) *Other issues*: A number of other ideas regarding improvement of care in the future emerged from the interviews. Though seemingly not closely related, they are grouped together in this last section of the results, since they were not fit for any of the sub-themes above. There was an opinion that future research on the topic is unnecessary. Also, it was argued that good research in this domain will only be possible if there is a clear definition and consensus about who is considered a bruxer. More research about the properties of splints would be welcome, so that their use becomes more evidence-based, rather than experience-based. *“Purely those splints, their shape, what is comfortable for the patient, what is optimal for the patient in terms of protection, because now I do something in an empiric way” [9]*. Finally, it was expressed that if patients were more informed and more understanding and accepting of the fact that bruxism may lead to a number of problems, the dentist’s job would become more pleasant.

## Discussion

The interviews showed that implantologists had a generally open attitude for performing implant-related treatments in patients with bruxism activity, and even though some complications might be expected, their extent is not such that bruxism is considered a contraindication. A number of studies have shown that bruxism (as diagnosed based on self-report and/or clinical examination, i.e., “possible and probable bruxism”) is associated with implant technical and, to some extent, biological complications.^3,16,17^ To our knowledge, there is no literature suggesting that bruxism is an absolute contraindication for dental implant treatments. Thus, it seems that both in daily practice and in research, implant complications can be expected in bruxers, and bruxism is not considered a contraindication for implant treatments per se.

Most implantologists had experienced technical complications of the implant-superstructure system, mainly porcelain fractures. This finding is consistent with bruxism-implant literature,^16^ and implant technical complication rates in general.^18^ Technical complications related to bruxism did not appear to be a large-scale burden for the implantologists in emotional and financial terms, but for the individual patient it was reported that this might be the case. In a small-scale prospective study, Spies et al.^19^ found patient satisfaction with function, esthetics, sense, speech, and self-esteem not to be affected by the occurrence of technical complications. Also, Bragger et al.^12^ found that taking care of biological and technical complications was related to low patient costs and visits in single-tooth replacements. However, Klinge et al.^20^ report that the matter of patient-related implant outcomes is “underexposed in research”, with the exception of mandibular overdentures.^20^ Taking also into account that bruxing individuals might have had multiple experiences with burdening dental complications prior to the start of the implant treatment, it is suggested that in this population, patient-related outcomes are further investigated.

Prominent controversy appeared to exist on the topic of bone loss and loss of osseointegration, with some implantologists arguing these can be etiologically related to bruxism, while others arguing against such a relation. Controversy on this topic was also found in other studies. Mattheos et al.^21^ investigated the attitudes of registered periodontists in Australia and the United Kingdom regarding the etiology of mucositis and peri-implantitis. The authors found that 15% of the Australian and 36% of the UK periodontists thought of adverse loading as an etiological factor. The difference between countries was significant.^21^ A similar study was carried out by Papasthanasiou et al.^22^ in the United States. Here, 71.8% of periodontists pointed adverse loading as an etiologic factor for peri-implant diseases.^22^ Though these studies were not specifically attributing adverse loading to bruxism, we can assume that bruxism can be a source of adverse biomechanical loading, therefore the outcomes of these studies are relevant for the bruxism-implant literature. Clearly, a wide diversity of opinions seems to exist not only within, but also between countries. Peri-implantitis is most probably the result of the interaction of many risk factors, with the importance of biomechanical overload still being controversial, and in need of further investigation.^20^ The above-mentioned variety of specialists’ opinions within and between countries most likely reflects the lack of unequivocal evidence on the relation between biomechanical loading and bone loss and/or loss of osseointegration, and a possible diversity in dental educational programs.^21^ From a patient’s perspective, it is not unimaginable that these diverging opinions may create confusion and subsequent distrust for the dental profession.

There were ample, diverging views on which features will lead to better treatment outcomes in bruxers. These included patient information and consent procedures, implant/superstructure material properties, occlusion and articulation patterns, skills of (general) dentists and technicians, post-treatment protection by splints, and post-treatment maintenance. Although variability of views was apparent, there was a general trend to focus on meticulous control of occlusion and articulation of the implant-supported superstructure. Views on the properties of correct occlusion of the implant fixed superstructure with its antagonist(s) varied from no contact at all, to contact only during biting hard in maximal occlusion, to no difference with contacts found in the natural dentition. To our knowledge, no literature exists that provides an evidence-based guideline for superstructure occlusion features, an observation that is not new.^23^ Occlusion and articulation can clinically be evaluated, are issues familiar to dentists, and can be modified at low costs; therefore, their significance in implantology practice should be further clarified.

As for implant-supported RPs, the general opinion of the interviewed implantologists was that these should not be worn during sleep. This reflects a protective measurement against SB, and requires patient compliance. If the prosthesis is not worn during sleep, wear of the mesostructure may occur, though the extent of this issue is not known. There was barely any mentioning of measurements that aimed to protect specifically from AB. Two interviewees described their approach for bruxist RP wearers. One preferred making the RP as mucosally supported as possible, so that pain will be felt when bruxing, an approach that may be helpful in cases of AB. This follows the rationale of “aversive conditioning,” that is, “the process in which an unwanted behavior is paired with a noxious or unpleasant stimulus, with the intention to reduce the undesired behavior.”^24^ The aversive conditioning approach has been discussed in bruxism literature, in the form of biofeedback techniques for the management for both awake and SB.^25^ To our knowledge, there is no evidence to support the effectiveness of this approach in implant dentistry. Mucosal pain was provoked in both conventional as implant-supported RPs during maximum bite force in the study of Fontijn-Tekamp et al.,^26^ though the conventional RP group presented significantly more pain than the implant-supported group. We were not able to obtain literature on the relation between bruxism and mucosal pain in implant-supported RP wearers. However, pain in the underlying soft tissue of conventional denture wearers was related to AB in the study of Piquero and Sakurai.^27^ The authors selected suspected awake bruxists based on soft tissue pain complaints, especially in the afternoon. They found that this group presented significantly more masseter muscle EMG activity that the control group (i.e., denture wearers without pain) during rest, and suggested that identifying AB is of great importance for the success of subsequent treatment of denture wearers.^27^ Similarly, Kumagai et al.^28^ showed that AB, as well as other factors (such as age, number of missing teeth, mucosal condition, mucosal damage, bone prominence), is an independent predictor of intensity and frequency of mucosal pain in the denture-bearing area of patients with partial removable dental prostheses. It would be of interest to investigate whether AB is related to mucosal pain in implant-supported RP wearers, and if so, if this mechanism can be used in treatment planning following the aversive conditioning paradigm. Meanwhile, we suggest that the clinician keeps an open eye for the possibility that otherwise unexplained mucosal pain complaints might be related to (undetected) AB, and/or SB, if the RP is worn during sleep.

In cases of a full upper RP against a fixed dentition in the lower jaw, another implantologist argued for making a rigid, metal reinforced base with soft artificial teeth. This way, the RP will not break under occlusal loading, but will rather be subject to wear and subsequent replacement of the artificial teeth. The implantologist mentioned high patient satisfaction with this approach. Clinical cases of protecting an implant-supported RP with metal parts from fractures have been published.^14,29^ In a 5-year prospective case series study, Boven et al.^30^ found acceptable/good outcomes in terms of implant survival, peri-implant bone level, probing depths, and peri-implant plaque, calculus, and bleeding indices for maxillary implant-supported overdentures opposed by (partial) natural dentitions, with the overdentures fabricated in a similar way as the implantologist described above.^30^ The authors also report good patient satisfaction at the end of the follow-up period.^30^ Thus, it is plausible that this is an effective treatment concept for the edentulous maxilla vs. (partially) dentate mandible, but the matter has not been researched in bruxing samples. We suggest that this be a topic of future research.

Skills of the dentist (e.g., in handling of implant components, such as tightening of screws) and of the dental technician (e.g., in designing the anatomical features of superstructures) involved in the treatment of the bruxer were repeatedly mentioned in the interviews as factors contributing to the success and complication rates. These factors have also emerged in other literature. In the study of Papathanasiou et al.,^22^ improper design of implant-supported restorations (i.e., a dentist and/or dental technician responsibility) was, among others, reported by registered periodontists in the United States as a possible etiological factor of peri-implantitis. Similarly, on the topic of peri-implantitis, Dawood et al.^31^ report that “peri-implantitis may be more frequently encountered when planning is poor, restorations are poorly designed and manufactured, implants and implant components are poorly engineered, and surgery poorly executed.” Heitz-Mayfield et al.^32^ report that, for the prevention of implant technical complications, careful treatment planning and handling of implant components is recommended. Spies et al.^19^ suggest that the more severe fractures of veneering ceramic of zirconia-based implant FDPs observed in their study “might be considered manual errors and not directly correlated with the composition of the veneering ceramic or the layering technique,” which, according to the authors, points to the importance of even the smallest omissions of the dentist or dental technician.

Post-treatment protection of the implant-superstructure complex during sleep by an occlusal splint was a standard advice by some, but not all interviewed implantologists. It makes logical sense that if the occlusal forces exerted when bruxing directly on the superstructure, and through this to the underlying implant, are responsible for subsequent complications, then placing a device on top of the superstructure that works as a wave breaker should solve many problems. This approach is commonly advised by expert opinions.^4,33,34^ However, the concept of the protective splint is not only not scientifically proven, but is seriously under-researched.^35^ As for the risks of wearing an occlusal splint, some precaution may be justified in patients suffering for obstructive sleep apnea (OSA), since it has been reported to aggravate their OSA condition.^13^ In other cases, though, having a patient wear a properly designed occlusal splint is not known to be able to cause any irreversible health damage. Therefore, the “better safe than sorry” approach currently followed is not to be blamed. However, if a splint in reality has no preventive value, then patients are confronted with unnecessary costs and the burden of having to be compliant with wearing it. Scientific evidence not only on the effectiveness of this intervention, but also on the features the ideal protective splint should have (in terms of material, thickness, etc.) is therefore necessary.

Several of the implantologists interviewed in this study mentioned the need for control of the occlusion and articulation features during the regular preventive check-ups at the general dentist (in the Netherlands, it is common practice that these check-ups take place every 6–12 months). This recommendation is driven by the thought that the occlusion changes over time, due to tooth wear and/or slight movements of the teeth. The implant-borne prosthesis will not move and is likely not to wear in exactly the same manner as the rest of the dentition, and therefore might come in the position of unwanted occlusion and articulation. The same recommendation is also found in the literature. Maintenance appointments with monitoring of the occlusion are suggested by Heitz-Mayfield et al.^32^ and Dawood et al.^31^

In our sample, bruxism is mainly diagnosed by clinical extraoral and intraoral examination, and to a lesser extent by patient anamnesis. Literature suggests starting with patient anamnesis (i.e., self-report and/or partner report) in order to assess the likelihood of a patient bruxing,^2^ that is, set the diagnosis of possible SB.^1^ It seems that in everyday practice implantologists tend to rely more on their clinical examination and tend to give little weight to what literature suggests should be the first diagnostic step in bruxism diagnosis. This may be explained by the fact that in everyday practice, experience has taught dentists that self-reported bruxism is of little validity, which is widely accepted in the literature too.^2^ The relation between anamnestic bruxism and a diagnosis based on anamnesis plus clinical examination has been the subject of investigation,^36^ and the concept of a graded bruxism diagnosis is already under revision since it was first published.^37^ The results of our study indicate that clinical examination for signs of bruxism may be more accepted and recognized by dentists for use in everyday practice. This also highlights a difference between the research and clinical practice world: if studies would use more clinical signs to diagnose bruxism instead of self-report, their results might be more translatable for everyday practitioners.

No uniform way to clinically diagnose bruxism evolved from the interviews. This might be related to the fact that even though standardized questionnaires do exist for assessing self-reported bruxism (e.g., Oral Behaviors Checklist;^38^ the BRUX scale^39^), this is not the case for a clinical diagnosis. Meaning, recommendations are given to look for clinical signs (such as impressions in lips, cheeks, tongue, tooth wear, etc.),^1,2,40^ but no standardized form or index exists in which dentists can score these signs (e.g., modified bleeding index^41^). This allows for subjective interpretations of which clinical signs to look for. Furthermore, an intuition-assisted diagnosis was not uncommon among the interviewed implantologists. Intuition is considered a key characteristic of clinical expertise, acquired by extensive learning.^42^ However, an intuitive diagnosis may not directly be considered valid.^42^ Such a diagnosis will rely on clinical suspicion, which arises when the clinician recognizes certain illness patterns.^42^ In low back pain literature, a “strong clinical suspicion” has recently been reported as having acceptably high diagnostic accuracy as a red flag for malignancy.^43^ We were not able to retrieve literature mentioning such a specific manner to diagnose bruxism. Therefore, we suggest that the extent of using this approach in bruxism diagnostics, and its validity be further researched.

Implantologists faced challenges related to the vagueness of the label of “who do we consider a sleep bruxer.” The issue of not everyone bruxing every night and not in the same intensity, that is, the time-variant nature of bruxism, was acknowledged. This has also been a topic of scientific research.^44,45^ SB diagnostic criteria have been established for PSG studies.^46^ These criteria are, however, not quite useful for daily practice, not only because PSG is unavailable in such a setting, but also due to the above-mentioned fluctuation in SB activity over time. We suggest that practitioners might be more helped if a diagnostic method existed that could identify the intensity of bruxism in a simpler manner, such as portable, user-friendly, single-channel EMG devices.

As for the mere importance of diagnosing bruxism, even though it was generally considered valuable to look for signs of bruxism, the significance of such diagnosis was questioned by the argument that there are more, and may be more important, reasons why implantological complications occur, and dentists ought to learn and be aware of them. Thus, practitioners seem not to attribute most of implant complications to bruxism. Literature suggests that the presence of bruxism may increase both implant failure rates, as the rates of implant technical complications, though other factors (e.g., material related) may also play a role.^47^ In their retrospective study, Chrcanovic et al.^48^ included 1406 patients who had received three or more implants in the course of 34 years (a total of 8337 implants), and investigated cluster behavior in implant failure (i.e., a patient having at least three dental implant failures) and related risk factors.^48^ Based on their analysis, the authors suggest that a cluster pattern among patients with implant failure is highly probable. Possible/probable bruxism emerged as an important risk factor for cluster failures, both at the patient (odds ratio (OR) 6.376) and the implant level (OR 5.2). Other potential risk factors also emerged from this study (shorter implants, turned implants, poor bone quality, age of the patient, intake of antidepressants and of medicaments to reduce gastric acid production, and smoking).^48^ Taking the observations of the interviewed implantologists and the above-mentioned literature into account, the picture arises that in daily clinical practice, bruxism is considered less of a problem than in published data. The nature of our study, that is, qualitative and not quantitative, allows for documentation of the opinions and attitudes of practitioners, but not for quantification of the extent in which they have actually dealt with, for example, bruxism-related complications. Therefore, our results are not directly comparable with those of other bruxism-implants literature. When attempting to use published literature as a basis, and the experiences of our interviewees as inspiration, we consider it plausible that: (a) bruxism is related to mechanical complications and implant failures, but (b) other risk factors do exist and an interaction with bruxism is likely, and (c) there may be a “high-risk” group of patients, in which bruxism, with or without the presence of other risk factors can be expected to cause more complications.

Additionally to what has been mentioned so far, we would suggest that in future research:

- the generic term of “bruxism” is avoided and the circadian manifestations, that is, awake and sleep bruxism are considered separately,
- a simple, chair-side, standardized tool is designed to diagnose sleep and AB at the possible/probable level in both research and clinical settings, as to make future research comparable and facilitate homogenous clinical practice,
- efforts are made for the design of user-friendly, and valid devices that will allow for a definite level of sleep and AB diagnosis in large-scale cohorts. The focus should not only be on the question *if* there is bruxism activity, but also on other aspects, such as type, intensity, and time intervals of activity,^49^
- the interaction between known risk factors for implant-superstructure complications is further investigated, as well as the possibility that risk profiles may exist both at the implant and the patient level, and
- special attention is given to the investigation of the effect of bruxism on bone loss and loss of osseointegration, since opinions in this field are highly divergent.

Several limitations of this study, and the measures taken to counter them, should be discussed. The prior development of domains, though on one hand valuable for the conduct of the study,^9^ may on the other hand been a source of bias, in terms of luring the interviewer to pay more attention to the discussion of these domains with interviewees, and not allowing for sufficient elaboration of new topics. Also, it could be possible that, to some extent, the interviewer’s own professional attitudes and experiences with the management of bruxism shaped not only the acquisition, but also the interpretation of data.^50^ In order to eliminate these undesirable influences, several measures were taken. There was a strong preference for open-ended questions, as to avoid guiding interviewees’ answers. However, in some instances this was not possible. For example, when the use of occlusal splints was discussed, the question “What sort of splint do you prefer?” was often asked. Though seemingly open-ended, the answer to this question would be either “hard” or “soft,” since these are the two types of occlusal splints mainly used in the Netherlands. Thus, this should be considered a closed-ended question. Furthermore, the interviewer made a careful effort to retain the role of a “curious listener,” by being alert for newly occurring concepts or ideas, and by exploring them further by asking appropriate probing questions. Interview questions were formulated in a neutral way, avoiding criticism on interviewee professional attitudes. In this aspect, though experienced in the field of bruxism, the interviewer is not clinically active in the field of oral implantology, a fact that was discussed with all interviewers prior to the start of the interviews. Thus, between interviewer and interviewees, there was no issue of comparing, nor judging of implantological treatment choices. As for issues related to bruxism, for example, the diagnosis, the professional background of both the interviewer (M.T.), as the second investigator analyzing the data (A.R.), might have had some influence in the interpretation of the data. In order to minimize this influence, a systematic, line-by-line analytic approach was maintained, and care was taken not to overemphasize or underemphasize any topic emerging from the interviews.

Certain issues regarding the sample characteristics should be mentioned. First, the all-male sample of this study was interviewed by a female investigator. It is unknown if this gender difference might have influenced the conduct of the interviews, or the interpretation of the data.^7^ Second, only 9, out of 169 who were initially approached, oral implantologists were included in this study. There was a very high number of invited oral implantologists who did not respond to the call for participation, possibly due to limited available time for participating in an interview. It is unknown if the oral implantologists who were eventually included in this study are different from their peers, in terms of professional experiences and attitudes. Sampling in this qualitative study continued until saturation of data was achieved, that is, until no new topics emerged from the interviews. Two extra interviews were conducted as to confirm this saturation, and thematic analysis provided a rich pallet of themes. If further investigation of the experiences and attitudes of oral implantologists is desired, the themes that emerged from this study could form a basis for the design of a quantitative study. An example hereof would be an online, questionnaire-based study, which could be less time-consuming for practicing oral implantologists, and may therefore acquire a larger sample size.

Finally, in this sample of nine oral implantologists, three reported not having followed any postgraduate education in the field of bruxism, in the 5 years prior to the conduct of the interview. The type and extent of continuing education might influence the attitudes and treatment choices of practitioners.^21^ Concepts about the nature of bruxism, that is, it being or not a pathological condition, as well as its diagnosis, are evolving.^37^ Therefore, it is suggested that ongoing education on this topic would be utterly beneficial for helping oral implantologists in handling based on state-of-the-art knowledge, and should be included in postgraduate educational resources.

## Conclusion

The findings of this study suggest that treating bruxing patients with dental implants in the practices of accredited oral implantology specialists in the Netherlands is generally well accepted. Complications can occur, and treatment planning should be careful, but bruxism is not considered a contraindication for implant treatment. Variability appears to exist in attitudes and opinions regarding bruxism diagnosis, the mechanism of biological complications and treatment planning approaches. The most divergent attitudes and opinions are those related to the associations between bruxism, bone loss, and loss of osseointegration.
